# Paths towards enhanced electrochemical CO_2_ reduction

**DOI:** 10.1093/nsr/nwz121

**Published:** 2019-08-23

**Authors:** Wenhao Ren, Chuan Zhao

**Affiliations:** School of Chemistry, University of New South Wales, Australia

Anthropogenic CO_2_ emissions engender a severe threat to the global ecosystem and consequently are a growing concern associated with traditional means of energy production. Direct electrocatalytic reduction of CO_2_ into energy-rich fuels and value-added chemical feedstocks provides a promising route to realize a carbon-neutral energy cycle, and at the same time, store the electricity generated from intermittent renewable-energy sources. Thermodynamically, the CO_2_ reduction reaction (CO_2_RR) suffers from a high energy barrier of CO_2_^•−^ formation (-1.90 V vs. SHE, pH = 7) and low selectivity due to the competing hydrogen evolution reaction (HER). Kinetically, multi-proton-coupled electron transfer steps upon CO_2_RR render sluggish reaction processes [1]. To tackle the above issues, significant progress has been achieved on the optimization of catalysts, products, and systems. The ultimate viability of this technology is contingent upon the exploitation of low-cost catalytic systems capable of providing high energy efficiency and conversion rate.

**Figure 1.**
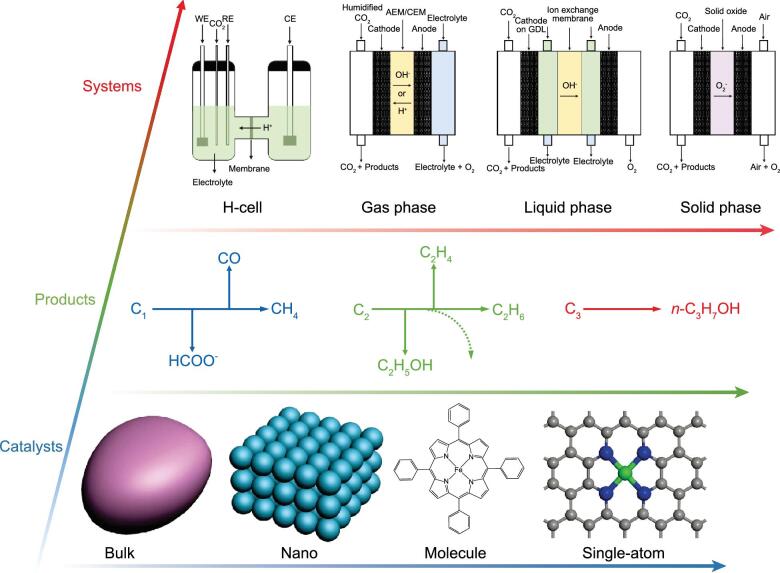
Path towards advanced electrochemical CO_2_ reduction from the perspectives of catalysts (bottom), products (middle) and systems (top).

Catalysts for CO_2_RR can be generally categorized into heterogeneous or molecular materials (Fig. 1, bottom) [2]. Nanostructured heterogeneous catalysts especially metal nanoparticles (e.g. Cu, Ag, Au, Sn) are among the most popularly utilized heterogeneous materials owing to the ease of synthesis and enhanced performance compared with bulk materials. Tuning the surface structure and composition of nanocatalysts is extremely crucial because most mass transfer processes occur on its surface. Although a vast number of researches have focused on modifying the nanomaterials from different angles, the inherent inactive properties of the atoms inside the material still limit its overall performance. Such a different behavior between surface and inside atoms also makes the mechanistic investigations difficult. Thus, the synthesis of heterogeneous catalysts with high atomic utilization and clear catalytic mechanism remains a great challenge. Molecular catalysts are typically comprised of organometallic complexes, their activity and selectivity are primarily determined by organic ligands coordinated with metal centers [3]. This family of catalysts possesses the advantages of highly exposed active sites and better-understood catalytic reaction mechanism. However, molecular catalysts tend to be plagued by inferior stability as they can easily aggregate to minimize the surface energy. It is envisaged that the rational design of molecular–heterogeneous hybrid catalysts for simultaneously achieving enhanced efficiency and stability will become an important area of research. Recently, single-atom catalysts (SACs) with isolated metal atoms anchored by covalent coordination are emerging as a new frontier in the catalysis community. Similar to molecular catalysts, SACs possess a well-defined and specific atomic structure which can offer high selectivity towards certain intermediates adsorption/desorption during CO_2_RR. Besides, their atomically dispersed nature can support a metal utilization up to 100%, resulting in high activity [4]. Metal-N-C (e.g. Fe, Ni, Co) based SACs have shown the state-of-the-art efficiency for CO_2_-to-CO production. Other SACs consist of noble metals (e.g. Au, Pd, Ru) are also promising and deserve more attention in the future research. Hence, the development of SACs paves a new way to design, as well as to understand, heterogeneous catalysis from the molecular angle and build a bridge between heterogeneous and homogeneous catalysis.

CO_2_RR can generate at least 16 different gas and liquid products depending on a variety of reaction pathways (Fig. 1, middle) [5]. C_1_ products including carbon monoxide and formate, via two electron transfer reactions, have been produced with high efficiency on a wide range of catalysts such as metal-N-C, Sn, In, Ag, Pd, etc. Other products such as methane, methanol, ethylene, ethanol and propanol, via multiple electron transfer reactions, have been generated with much lower Faradic efficiencies typically on Cu-based electrodes. This mainly stems from the competing formation of C–C, C–H and C–O bonds and the additional reaction barrier associated with a key step regarding C–C bond formation. Whilst the multi-carbon products have a wide application market, in part due to their high energy density, their production efficiency via CO_2_RR need to be improved to become economically viable. To achieve this goal, the rational design and synthesis of catalysts, especially for Cu-based materials, with desired electronic and morphological properties are pivotal [6]. Different from the well-established formation mechanism for C_1_ products, the reaction pathways for C_2+_ products are more complex and highly dependent on the catalyst surface and intermediates. Advanced approaches including *in situ* characterizations (e.g. X-ray absorption spectroscopy and infrared spectroscopy), computational simulation, and isotope labeling for the in-depth understanding of the reaction pathways are particularly desired.

To date, most CO_2_RR researches have been focused on the design and synthesis of catalysts, during which the electrochemical behaviors were studied in aqueous systems using an H-cell electrolyzer. From a practical perspective, however, these testing systems have many intrinsic limitations that must be optimized. Most notably, the poor solubility of CO_2_ (∼34 mM at 25°C) in aqueous electrolytes is an intrinsic limitation for achieving high energy efficiency and conversion rate. Non-aqueous solvents, such as ionic liquids, can offer much higher CO_2_ solubility than water. Further, it has been revealed that certain ionic liquids can serve as a co-catalyst for CO_2_RR and facilitate the formation of intermediate products [7]. Mixed electrolyte systems containing aqueous and non-aqueous solvents are therefore a promising area of future study for improving electrochemical CO_2_RR. Other important factors and potential avenues of research include cation and anion effects, ionic transport properties, and pH.

In addition to the electrolyte, the traditional H-cell device typically operates with current densities less than 100 mA cm^−2^. Such low current densities are ill-suited to industrial manufacturing (>300 mA cm^−2^). Thus, learnt from abundant knowledge of fuel cells and water electrolyzers, an alternative approach is to use flow cells including gas-, liquid-, and solid-phase prototypes for scaled-up implementation (Fig. 1, top) [8]. Gas-phase electrolyzer has low ohmic loss, while the competition of HER and the hydration of membrane are the intrinsic drawbacks hindering its long-term operation. Liquid-phase electrolyzer can support high current density, but the ohmic loss and the flooding risk should be addressed before its application. Solid-oxide electrolyzer consists of a solid cathode, anode, and electrolyte. They combine electrocatalysis with high temperatures (>873 K) to generate C1 gas products (e.g. CO or CH_4_) at current densities typically in the order of A cm^−2^ from CO_2_ and H_2_O/H_2_ feedstocks. Although this technology operates at high current densities and low cell voltages, the limited product range and extreme temperature requirement restrict its widespread application. Gas diffusion electrodes (GDEs) are considered as the heart of most flow cells, and consist of a hydrophobic gas diffusion layer with a catalyst layer deposited on one side. The GDE design possesses several significant advantages: (i) dramatically enlarged catalyst/electrolyte/gas three-phase boundaries for CO_2_RR to occur; (ii) fast gas phase transport of reactant CO_2_ and efficient bubble/water removal; (iii) protection of the catalyst layer from leaking or erosion caused by flows or other factors; (iv) use of potassium hydroxide instead of buffer solution as the electrolyte to achieve higher CO_2_RR efficiency. Based on this design, the state-of-the-art Faradaic efficiency of the C_2_ product (C_2_H_4_) on a Cu catalyst reaches ∼60% with a full-cell energy efficiency of 34% [9].

Currently, most researches use pure CO_2_ gas for CO_2_RR investigation, while the capture and enrichment of CO_2_ gas require additional cost especially from the atmosphere. Another highly desirable application of CO_2_RR is the direct conversion of CO_2_ from industrial waste gases into valuable products [10]. To this end, exploiting efficient catalysts for the electroreduction of low concentration CO_2_ (∼10%) is significant yet rarely investigated. Apart from the CO_2_, industrial exhaust may also contain S-, N-, and O-based gases, which may result in other competing reactions such as O_2_ reduction reaction (ORR). The interactions among CO_2_RR, HER and ORR therefore need to be systematically studied from both theoretical and experimental standpoints. Currently, industrial electrochemical CO_2_RR may not be economically advantageous for producing carbon-based products compared with using traditional fuels as the chemical feedstock. Nevertheless, the realization of efficient electrochemical CO_2_RR on large scale is beneficial, both as a mean for renewable energy storage and for addressing urgent environmental issues associated with CO_2_ emission. Despite breakthroughs on various catalysts, the fundamental bottleneck of electrochemical CO_2_RR towards industrialization lies in system-level optimization. Solely focusing on either catalysts, products, electrolytes, or cell design is unlikely to be effective; an optimization among all the components is ultimately required.
